# Comparison of Bond Strength of Metal and Ceramic Brackets Bonded with Conventional and High-Power LED Light Curing Units

**Published:** 2016-11

**Authors:** Javad Chalipa, Yasamin Farajzadeh Jalali, Fatemeh Gorjizadeh, Pedram Baghaeian, Mohammad Hashem Hoseini, Omid Mortezai

**Affiliations:** 1 Assistant Professor, Dental Research Center, Dentistry Research Institute, Tehran University of Medical Sciences, Tehran, Iran; Department of Orthodontics, School of Dentistry, Tehran University of Medical Sciences, Tehran, Iran; 2 Assistant Professor, Department of Orthodontics, School of Dentistry, Ilam University of Medical Sciences, Ilam, Iran; 3 Assistant Professor, Department of Orthodontics, School of Dentistry, Yasuj University of Medical Sciences, Yasuj, Iran; 4 Private Practice, Tehran, Iran; 5 Assistant Professor, Department of Orthodontics, Qazvin University of Medical Sciences, Qazvin, Iran

**Keywords:** Orthodontic Brackets, Shear Strength, Light-Curing of Dental Adhesives

## Abstract

**Objectives:**

The aim of this study was to evaluate the effect of conventional and high-power light emitting diode (LED) light curing units on shear bond strength (SBS) of metal and ceramic brackets to tooth surface.

**Materials and Methods:**

Forty sound bovine maxillary central incisors were used for the study. The teeth were divided into four groups (n=10). Teeth surfaces were etched with 37% phosphoric acid for 20 seconds. After applying a uniform layer of adhesive primer on the etched enamel, composite was placed on the base of brackets. The samples were light cured according to the manufacturer’s instructions and thermocycled. The SBS was measured. The failure mode was scored using the adhesive remnant index (ARI).

**Results:**

The mean SBS of samples in groups A (high-power LED, metal bracket), B (high-power LED, ceramic bracket), C (conventional LED, metal bracket) and D (conventional LED, ceramic bracket) was 23.1±3.69, 10.7±2.06, 24.92±6.37 and 10.74±3.18MPa, respectively. The interaction effect of type of LED unit (high-power/conventional) and bracket type on SBS was not statistically significant (P=0.483). In general, type of LED unit did not affect SBS. Type of bracket significantly affected SBS (P<0.001). The ARI score was not significantly influenced by the interaction between the type of LED unit and bracket.

**Conclusions:**

The obtained SBS is the same for both bracket types by use of high-power and conventional LED light curing units. Regardless of the type of LED unit, SBS of ceramic brackets was significantly lower than that of metal brackets.

## INTRODUCTION

Appropriate bond strength between bracket and tooth surface is one of the most important aspects of orthodontic treatments [1,2]. Bonding of orthodontic brackets to enamel started in the mid 1960s [3,4]. Only auto-polymerizing materials were available at the time. Bonding of orthodontic brackets with visible light-cure adhesives was first reported by Tavas and Watts [5]. The light-cure adhesives were widely accepted due to their advantages in comparison with other chemical-cure adhesives. These advantages include high primary bond strength, better physical characteristics because of air inhibition phenomenon, user friendly application, extended working time for precise bracket placement and better removal of adhesive excess; but they have three major disadvantages namely being time-consuming, hindering light transmission and polymerization shrinkage [6,7]. Since then, several new methods using different composites and light-curing units have been introduced for this purpose. The halogen lamp, also known as quartz halogen and tungsten halogen lamp, has been used as light-curing unit for many years [8,9], and is the most common source of visible blue light for dental applications. This lamp contains a blue filter to produce light of 400–500 nm wavelength [10]. The wide spectrum of action, easy use and low-cost maintenance are the most favorable characteristics of halogen light curing systems [9]. Despite their popularity, halogen light curing units have several disadvantages. For example, their light power output is 1% of the total electric energy consumed [11,12]. Moreover, the lamp, reflector and filter wear out gradually [13]. Halogen bulbs have a restricted useful lifetime of about 40–100 hours [13,14]. The power density of light curing unit decreases with increase in distance. The other drawback of application of halogen bulbs is prolonged curing time [15,16]. Over the past several years, other light sources such as xenon plasma arc, argon laser, and light-emitting diodes (LEDs) have been introduced in orthodontics [17]. According to the results of previous studies [1,18–20], the shear bond strength (SBS) values of orthodontic brackets in curing with halogen lamps and plasma arc are the same but plasma light reduces curing time per tooth from 20–40 seconds to two seconds. Also, argon laser curing unit provides better SBS than halogen lights. But xenon plasma arc and argon laser are too expensive [18]. Mills [19] introduced LED light curing units as a polymerizing light source in 1995. At present, LED sources are among the most reliable light source categories for bracket bonding [8,20]. Light cure resins set when irradiated with light at wavelengths of 460nm and 480nm in the blue end of visible spectrum with an intensity of 300mW/cm ^2^ [21]. Also, LED is an effective transducer of electrical power into visible blue light and does not produce a lot of heat [8]. The advantages of LED light curing units include lifetime of several thousand hours without significant degradation of light flux over time, resistant to shock and vibration and no need for filter to produce blue light [22–24]. Moreover, LED light curing units consume little power and can be run on rechargeable batteries, allowing them to have a lightweight ergonomic design [25]. The new LED curing units were launched simultaneously with the advancement of technology. First, these curing units generated light with an intensity of approximately 800–1000YmW/cm ^2^ , reducing the required light exposure time to 10 seconds [26,27]. Currently, some high-power LED curing units are able to emit light radiation with intensity of 1600–2000YmW/cm ^2^ , allowing shorter exposure times of six seconds for metal brackets [28]. In this study, the effect of conventional and high-power models of LED units on SBS of metal and ceramic brackets to tooth surfaces was evaluated.

## MATERIALS AND METHODS

Forty sound bovine maxillary central incisors were used in this study. After extraction, the teeth were cleaned and immersed in 0.5% chloramine solution at 4°C for one week. They were divided into four groups of 10 teeth in each group. Next, teeth surfaces were etched with 37% phosphoric acid (Reliance; Itasca, IL, USA) for 20 seconds. After etching, the teeth were washed with water spray for approximately 10 seconds. The sample size (n=8 minimum samples for each group) was calculated with a power analysis in order to provide a statistical significance of alpha=0.05 and a standard deviation of 4.2 MPa using Minitab software. Sampling method in the study was consecutive. Bracket model and the type of light curing unit used for teeth were determined randomly.

**Fig. 1:**
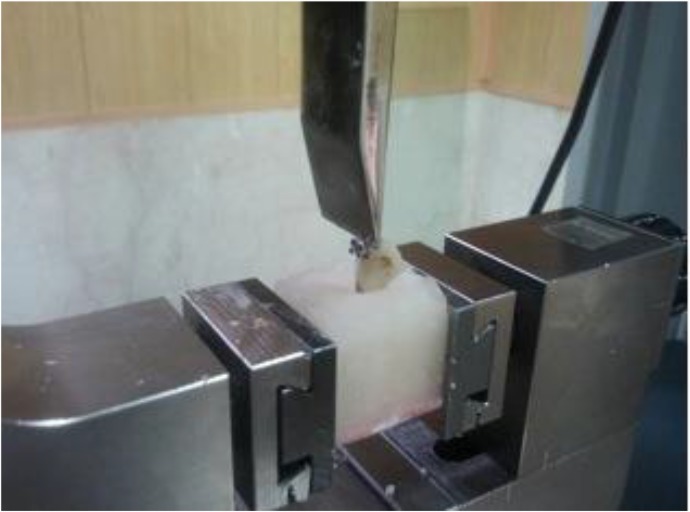
Testing shear bond strength of metal brackets

Group A: After checking correct conditioning of the enamel, metal brackets (American Orthodontics, Sheboygan, WI, USA) with a nominal base area of 11.3mm ^2^ were bonded with Transbond XT (3M ESPE, St. Paul, MN, USA), applying a uniform layer of adhesive primer on the etched enamel, and resin cement on the base of brackets. Brackets were placed in place and were pressed against the surface of the tooth. Excess cement was carefully removed with a dental probe, and the adhesive was high-power light-cured (2700mW/cm ^2^ ; Dentlight LLC, Plano, TX, USA) for four seconds (two seconds from mesial and two seconds from distal).
Group B: Ceramic brackets (American Orthodontics, Radiance Plus, Sheboygan, WI, USA) with a nominal base area of 15.1mm ^2^ were bonded to the etched enamel and other steps were performed similar to group A. The adhesive was high-power light-cured for three seconds (1.5 seconds from mesial and 1.5 seconds from distal).
Group C: Metal brackets (American Orthodontics, Sheboygan, WI, USA) with a nominal base area of 11.3mm ^2^ were bonded to the etched enamel and other steps were performed similar to other groups. The adhesive was light-cured conventionally (600 mW/cm ^2^ ; Dr’s light, Good Doctors Co., Ltd., Incheon, South Korea) for 20 seconds (10 seconds from mesial and 10 seconds from distal).
Group D: Ceramic brackets (American Orthodontics, Radiance Plus, Sheboygan, WI, USA) with a nominal base area of 15.1mm ^2^ were bonded to the etched enamel and other steps were performed similar to other groups. The adhesive was light-cured conventionally for 20 seconds (10 seconds from mesial and 10 seconds from distal). The samples were mounted in a metal mold containing auto-polymerizing acrylic resin and thermocycled for 2,500 cycles between 5–55°C for 20 seconds at each temperature with 20 seconds of transfer time. Rectangular wires were used to match the central alignment of teeth in acrylic resin. All samples were subjected to SBS test in a universal testing machine (7060; Zwick Roell, Ulm, Germany) at a crosshead speed of 3 mm/minute (Fig. 1).

The results were obtained in kilogram-force, converted to Newtons and then to megapascals (MPa). After failure, the samples were observed under a stereomicroscope (SMZ 800; Nikon, Tokyo, Japan) at ×20 magnification to score the amount of remaining adhesive using the adhesive remnant index (ARI) [29]: 0=No adhesive remained on the tooth; 1=Less than 50% of adhesive remained on the tooth; 2=50% or more of the adhesive remained on the tooth surface; 3= 100% of the adhesive remained on the tooth, with a distinct impression of bracket mesh, corresponding to failure at the bracket-adhesive interface. Data were statistically analyzed using SPSS version 22.0.0 (SPSS Inc., Chicago, IL, USA).

The mean, standard deviation, minimum and maximum values of SBS of metal and ceramic brackets to tooth surfaces using two models of light-curing units were computed and reported. The SBS data were analyzed using one-way ANOVA, followed by Tukey’s post hoc test. Failure mode data were subjected to Kruskal-Wallis nonparametric test, followed by LSD post hoc test. Statistical significance was set at alpha=0.05.

## RESULTS

According to the results presented in Table 1, the mean SBS of samples in groups A, B, C and D was 23.1±3.69, 10.7±2.06, 24.92±6.37 and 10.74±3.18MPa, respectively.

**Table 1:** The shear bond strength values (in megapascals) of metal and ceramic brackets to tooth surfaces using high-power and conventional LED light curing units

** Group **	** Curing time (s) **	** Mean **	** Standard deviation **	** Min **	** Max **
** A **	4	23.01	3.69	17.8	28.34
** B **	3	10.7	2.06	12.8	14.06
** C **	20	24.92	6.37	15.22	32.34
** D **	20	10.74	3.18	4.71	15.24

**Fig. 2:**
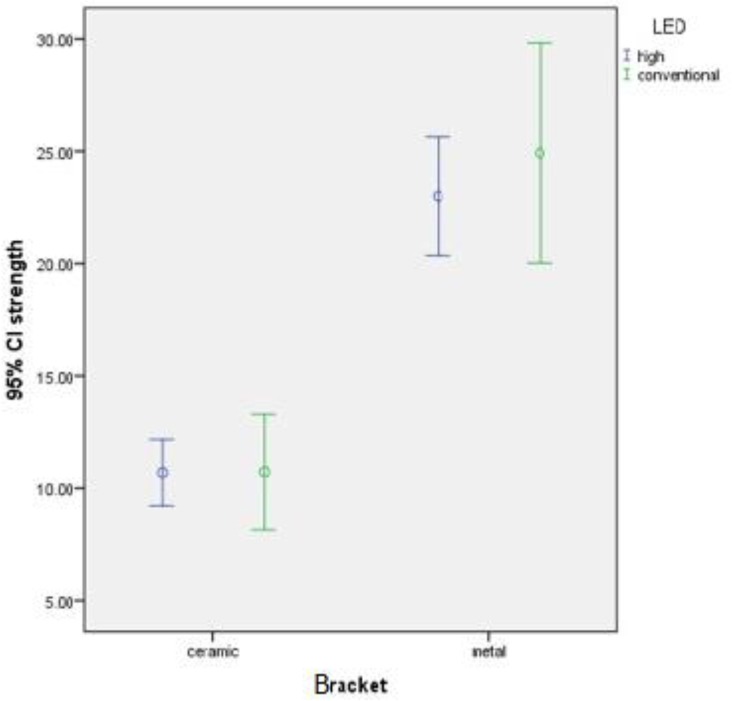
The mean and 95% confidence interval of shear bond strength of metal and ceramic brackets to tooth surfaces with high-power and conventional LED light curing units

The mean and 95% confidence interval of SBS of metal and ceramic brackets to tooth surfaces using high-power and conventional LED light curing units are shown in Figure 2. Two-way ANOVA revealed a statistically significant difference in SBS among the groups (P=0.003). According to homogeneity of variances (Leven’s test, P=0.131), Tukey’s HSD test was applied for multiple comparisons.

The interaction effect of type of LED unit (high-power and conventional) and bracket on SBS was not statistically significant (P=0.483). In general, type of LED unit did not affect the SBS (P=0.467). According to the results of this study, type of bracket significantly affected the shear bond strength (P<0.001). Distribution of failure mode in the four experimental groups is presented in Table 2. The ARI score was not significantly influenced by the interaction effect of the type of LED (high-power and conventional) and bracket (P>0.05). The remaining adhesive in all ceramic brackets was less than 50%; while ARI scores in metal brackets were more diverse. There were no significant differences between ceramic and metal brackets in ARI scores (P>0.05).

**Table 2:** Descriptive data of adhesive remnant index (ARI) scores in the groups

** Bracket **			** ARI **				

			0	1	2	3	Total
** Ceramic **	LED	High-power	0	10	0	0	10
		Conventional	1	9	0	0	10
		Total	1	19	0	0	20
** Metal **	LED	High-power	3	3	2	2	10
		Conventional	3	3	3	0	9
		Total	2	6	9	2	19
** Total **	LED	High-power	2	13	3	2	20
		Conventional	1	12	6	0	19
		Total	3	25	9	2	39

## DISCUSSION

In this study, we used SBS test, which is commonly used and has acceptable repeatability. This method has a high similarity to oral environment in terms of applied forces to samples. It has been discussed that different laboratory tests such as shear, compressive and tensile bond strength tests yield variable results and their findings cannot be compared [29,30].

Oral environment is complex and cannot be exactly simulated in vitro due to the presence of variables like saliva, diet and patient’s habits. Thus, almost all studies in this field use aging protocols. Applying thermal cycling to samples is one common method to simulate oral environment. In this study, all samples were thermocycled for 2,500 cycles between 5–55°C for 20 seconds. Previous studies have shown that thermocycling and water storage can affect the SBS [31,32]; without an aging protocol, only primary SBS can be measured. Daub et al. [33] reported that 500 thermal cycles would decrease the SBS by about 16.7%. Although Arici and Arici [34] reported a 5.7% decrease in SBS after 200 thermal cycles.

However, some studies have demonstrated that there is no difference in SBS with or without thermocycling [35–38]. Cerekja and Cakirer [39] showed that there is no variation in SBS of metal brackets bonded to teeth using different light curing units, with or without thermocycling.

The SBS of brackets has been widely studied. Ideal bracket bonding should hold the attachment on tooth surface during the entire period of treatment and should withstand both orthodontic and masticatory forces. At the end of treatment, brackets should be easily detached from the teeth without damaging the enamel.

In orthodontic treatment, one of the most important factors for bond strength of light-cure adhesives is the curing method. Previous studies have shown that LED light curing units can polymerize adhesives as efficient as halogen devices [15,39]. Despite lower radiation ability of LED units compared to halogen devices, LED units are more efficient in terms of adequate light transfer [11].

The advantages of LED units to halogen and plasma arc units include wireless system, lighter weight, smaller design and lifespan of 10,000 hours [22]. Plasma arc units are two to three times more expensive than halogen devices. Also, LED units are more expensive than halogen devices but cheaper than plasma arc. It has been reported that six seconds of curing by plasma arc creates bond strength as high as 40 seconds of radiation by halogen devices [40]. The high-power LED unit that was used in this study was cheaper than plasma arc but more expensive than conventional devices and due to remarkable reduction in chair time, the higher price is justified.

Swanson et al. [41] showed that 40 seconds of curing by LED units results in a stronger bond, but 20 seconds of curing time also creates a bond strength higher than the required amount (>8MPa). In this study, 20 seconds of radiation was considered for the conventional unit for both bracket types and four seconds of curing for metal brackets and three seconds for ceramic brackets by high-power LED unit were considered. The mean bond strength for ceramic brackets was in the required range for both LED units; while the bond strength of metal brackets was higher than required. The lower bond strength of ceramic brackets could be due to the type of ceramic brackets used in this study. These types of brackets have no base design for micromechanical retention and also to reduce chemical bond strength; thus, chemical bond would only take place at the center of bracket base and this theorem was well observed when ARI scores were evaluated.

There is little data about new high-power LED units. Ward et al, [42] in a randomized split-mouth design study demonstrated that there was no difference in bond failure rate of brackets bonded by standard-intensity (1200 mW/cm ^2^) and high-intensity (3200 mW/cm ^2^) LED units. They reported that six seconds of curing by a high-power LED unit is comparable with 20 seconds of radiation with an ordinary LED unit. High-intensity devices must be used with care to avoid heat trauma to the pulp complex [43]. In this study, we did not check the thermal changes caused by light curing. According to Ramoglu et al, [43] in their in vitro study, the most powerful LED unit (3200mW/cm ^2^) with three seconds of irradiation caused the lowest rise in temperature in comparison to other LED units with lower powers (1000 mW/cm ^2^ , 1200 mW/cm ^2^ and 1400 mW/cm ^2^) and longer time of curing (15 seconds, 10 seconds and eight seconds) and also, all light curing units that were assessed in their study generated temperature rises within the safe range for dental pulp. Therefore, high-power LED units can be used with confidence knowing that they do not cause thermal damage to dental pulp.

In this study, there was no difference in ARI scores between LED units. This finding is consistent with the results of other studies that compared different types of curing units and reported no difference in ARI scores [35, 39, 43].

Also, there was no difference in ARI scores of the two bracket types; however, the adhesive remnant pattern for all samples of ceramic brackets except one showed score 1 that means less than half of adhesive remained on tooth surface; but in use of metal brackets, all scores were found in samples. This could be because of base design of ceramic brackets, which was mentioned earlier. Adhesive bond failure occurred at the center of bracket base, while bond failure was cohesive at the peripheral parts.

## CONCLUSION

The SBS of both brackets (metal and ceramic) by use of high-power and conventional LED units was the same. Therefore, using high-power LED units with shorter curing time is suggested. There was no difference in ARI scores with regard to bracket types or LED units used.
